# Artistic sports activities effectiveness for enhancing students’ academic performance among left-behind children: mediating effects of loneliness

**DOI:** 10.3389/fpsyg.2024.1366501

**Published:** 2024-05-06

**Authors:** Yutao Zhou, Francesco Vincenzo Ferraro, Chengwen Fan

**Affiliations:** ^1^Physical Education Institute, Hunan University of Technology, Zhuzhou, China; ^2^Hunan Research Centre for Excellence in Fitness, Health and Performance, Zhuzhou, China; ^3^School of Human Sciences, University of Derby, Derby, United Kingdom

**Keywords:** artistic sports activities, left-behind children, academic performance, student’s academic self-efficacy, self-esteem, loneliness

## Abstract

**Introduction:**

Numerous studies within the school and academic contexts have underscored the profound impact of psychometric variables such as academic self-efficacy, self-esteem, and loneliness on academic achievement among children. Although physical activities and dance practices are known to bolster academic self-efficacy and enhance academic outcomes, the effect of Artistic Sports Activities on these psychological determinants among left-behind children (LBC) in rural schools remains underexplored.

**Method:**

This study was conducted from September 2020 to January 2022 among 405 LBCs aged 9 to 13 from six randomly selected primary schools in Hunan Province, China. Schools were chosen in collaboration with the Hunan Women and Children’s Federation, ensuring informed consent through stakeholder informational sessions. The study employed rigorous sampling and data analysis methods, including the Shapiro–Wilk test for normal distribution and Cronbach’s alpha for reliability, alongside Pearson correlation, independent and paired t-tests, and multiple linear regression analyses to investigate the effects of Artistic Sports Activities on psychometric variables and academic performance among LBCs. Data collection involved standardized questionnaires assessing academic self-efficacy, self-esteem, and loneliness before and after intervention.

**Results:**

Findings indicate that Artistic Sports Activities significantly improved academic self-efficacy and self-esteem and reduced loneliness, leading to enhanced academic performance. Notably, loneliness was identified as mediating the relationship between academic self-efficacy and self-esteem among LBCs.

**Discussion:**

The findings highlight the critical role of integrating Artistic Sports Activities in educational frameworks to bolster psychological and academic outcomes for LBCs. The study reveals the intricate interplay between loneliness, self-esteem, and academic self-efficacy, underscoring the necessity for targeted educational interventions.

## 1 Introduction

In China, large numbers of rural residents are migrating to urban areas for job opportunities (Gu, 2022), resulting in the emergence of Left-Behind Children (LBC), kids under 18 with one or both parents working away, leaving them with grandparents or local communities (Chen et al., 2022). The school environments of these LBCs reflect significant challenges rooted in psychosocial factors. Extended parental absence correlates with adverse educational outcomes such as increased school dropouts (Li and Hu, 2021), bullying in school (Zhang et al., 2022), high suicidal thoughts (Zhou et al., 2022), aggressive behaviors in school (Yu et al., 2022), higher levels of antisocial behavior (Liu et al., 2022), loneliness (Wang Q. et al., 2021), reduced academic performance (Mao et al., 2020), lower self-esteem (Zhou et al., 2022) and lower self-efficacy (Liang et al., 2018).

From a the school and academic contexts, it’s imperative to understand the interplay of psychometric variables like academic self-efficacy, self-esteem, and loneliness in shaping a student’s educational journey (Neroni et al., 2022). As shown in various studies, these psychological determinants are pivotal in predicting academic outcomes. For instance, Bhatt and Bahadur (2018) highlighted that higher levels of academic self-efficacy among students increased motivation and resulted in higher academic performance, while Huang (2011) found a positive association between self-esteem and academic performance, as well as Honicke and Broadbent (2016) emphasized that academic self-efficacy has a strong moderated positive correlated with academic performance. Moreover, the adverse effects of loneliness on students’ well-being and academic progress have been documented by Qualter et al. (2015). In a pivotal finding, Zhou et al. (2023) discovered that improving self-esteem can increase academic self-efficacy, thereby enhancing the academic performance of LBCs. These insights underscore the significance of addressing psychometric variables in educational settings to foster a conducive learning environment (Table 1).

**Table 1:** Overview of psychometric variables influencing academic performance.

Psychometric Variable	Definition	Influence on academic performance
Students’ Academic Self-Efficacy	A student’s belief in their ability to meet the demands of their academic environment (Fife et al., 2011).	Significant influence on predicting academic resilience (Bandura, 1997). Facilitates the development of academic goal-setting skills (Locke and Latham, 2002) and impacts academic performance (Honicke and Broadbent, 2016).
Self-Esteem	A psychological state corresponds to a sense of integrity in understanding one’s capabilities and value (Saha, 2018).	Mediates the relationship between parent–child relationships and academic stress (Mulyadi et al., 2016). Strongly correlates with academic performance and teacher support (Liu et al., 2019).
Loneliness	An unpleasant emotion of discrepancy between desired and experienced interpersonal relationships (Perlman and Peplau, 1982), is typically experienced by LBC (Shen et al., 2015).	Associated with a lack of family connections (Cacioppo et al., 2015), and correlated with individual problems among LBC, such as emotional problems (e.g., social anxiety) (Li et al., 2020), psychological problems (e.g., self-esteem) (Wei and Huang, 2019), and education-related problems (e.g., student academic performance or achievement) (Wang Y. et al., 2021).

Within the school and academic contexts, the triad of academic self-efficacy, self-esteem, and loneliness has been recognized as crucial determinants of students’ academic performance. However, the intricate relationships among these psychometric variables, especially the mediating role of loneliness between self-esteem and academic self-efficacy, remain uncharted for left-behind children (LBCs).

According to Bandura’s Social Cognitive Theory (Bandura, 2012), individuals’ mental states are influenced by combining self-influence factors and social systems (Bandura, 1977). Regarding self-influence factors, Bandura’s Social Cognitive Theory highlights the significance of self-efficacy, which refers to an individual’s belief in their capabilities to organize and execute actions to achieve desired results (Bandura, 1997). In the academic context, researchers often use academic self-efficacy to mean the level of self-efficacy that learners judge in themselves about their abilities to achieve educational goals (Morales-Rodríguez and Pérez-Mármol, 2019). Affuso et al. (2022) highlighting that high self-efficacy improves children’s academic performance. Another self-influence factor is self-esteem (Bhatt and Bahadur, 2018), which is included in Abraham Maslow’s theory of “Maslow’s Hierarchy of Needs.” Maslow and Lewis (1987) suggest that if self-esteem needs are unmet, individuals cannot grow, develop, or achieve their self-goals, including children’s academic performance (Noronha et al., 2018).

When considering the social systems, Bronfenbrenner’s ecological systems theory (Bronfenbrenner, 1992) underscores the family and peers’ pivotal roles in influencing children’s development, especially in adversities (Ren and Li, 2020). This emphasis is critical when a child’s primary microsystem fails to provide the needed psychological support, spotlighting the importance of other microsystems in such instances (Hertler et al., 2018). For children from left-behind backgrounds (LBCs), the absence of parental and peer support markedly increases their risk of social anxiety and loneliness (Cavanaugh and Buehler, 2016). Theoretical insights suggest that the quality of interactions within these social systems–family and peers–can profoundly affect self-influencing factors, such as self-esteem and academic self-efficacy. Loneliness, significantly shaped by these interactions, emerges as a critical factor impacted by family and peer dynamics. Meanwhile, Fuentealba-Urra et al. (2022) highlighted the importance of considering the relationship between the type and context of physical activity in adolescents. This underscores the theory’s broader implication that nurturing relationships in these systems can mitigate negative emotional states and enhance psychological resilience in children.

In the diverse range of school-based activities, Wang et al. (2024) revealed that school-based physical activity significantly influences children’s self-identity and reduces social anxiety, also artistic sports activities have uniquely beneficial, which include artistic elements like music, choreography, and costumes, they aim to boost the sport’s esthetic value and provide an audio-visual and postural expression of fitness and esthetic appreciation (Qi, 2018). This blend distinguishes artistic sports from their non-artistic counterparts by fostering physical and mental growth, offering a distinctive pathway to bolster children’s psychological health (Zhang, 2009). Jianshe Zhou (2020) notes the unique potential of these activities, beyond what conventional activities can achieve, to fortify psychological well-being. Disciplines such as figure skating, aerobic gymnastics, dance sports, rhythmic gymnastics, and synchronized swimming (Qi, 2018) play pivotal roles. Specifically, Latino Dance has been found to reduce loneliness (Hong-gang Shan and Yu, 2011) and significantly enhance academic self-efficacy (Zhou et al., 2023), diminish anxiety (Shan and Yu, 2011), lessen self-accusation tendencies and improve both self-efficacy (Hui and Junyu, 2014) and self-esteem among LBC students (Zhou et al., 2023).

Therefore, this study aims to investigate whether artistic sports activities interventions (Aerobic Gymnastics and Latin American dance) can enhance the academic performance of LBC (improve students’ academic self-efficacy and self-esteem and reduce loneliness). Four research hypotheses will be verified in this research: Hypothesis 1 (H1): Artistic Sports Activities interventions, specifically Aerobic Gymnastics and Latin American dance, will increase academic self-efficacy among LBCs. Hypothesis 2 (H2): Participation in Artistic Sports Activities will improve self-esteem levels among LBCs. Hypothesis 3 (H3): Engaging in Artistic Sports Activities will reduce feelings of loneliness among LBCs. Hypothesis 4 (H4): Loneliness will mediate the relationship between academic self-efficacy and self-esteem among LBCs, such that reduced loneliness associated with participation in Artistic Sports Activities will improve both self-esteem and academic self-efficacy.

## 2 Methods

### 2.1 Design

This study adopted a research design to investigate the impact of physical education (PE) on the holistic development of left-behind children (LBCs) in primary schools across Hunan Province, Southern China. The suitability of this design was chosen to match the study’s research aims, which aimed to explore the associations between artistic sports intervention and various psychological outcomes in a specific educational context, providing valuable insights into the potential benefits of PE for child development, particularly among LBCs.

### 2.2 Participants

Between September 2020 and January 2022, this study was conducted in six randomly selected primary schools in Hunan Province, Southern China. Southern China. Hunan Province, known for its diverse educational landscape and significant number of ‘left behind’ children due to urban migration, provides a unique context for studying the impact of physical education (PE) on child development. The China PE primary curriculum, which emphasizes the holistic development of students through physical activities, sports, and health education, plays a crucial role in the well-being of children, particularly those left behind by migrating parents. These schools were selected using a list provided by the government-sponsored Hunan Women and Children’s Federation. Before the commencement of the study, the research team established a strong relationship with the school principals, obtaining their permission to proceed. Informational sessions were then conducted with teachers, parents, and guardians to clearly understand the research objectives, procedures, and potential benefits for the left-behind children. Informed consent forms were distributed to parents or guardians, emphasizing the voluntary nature of participation, and contact information was provided for any questions or concerns. Furthermore, interactive activities and workshops were organized in collaboration with the school staff to foster a supportive and inclusive environment, ensuring the active engagement and involvement of the left-behind children in the study.

Four hundred eighty-six (486) 9 to 13-year-old LBCs were invited to participate in the study. Each LBC voluntarily registered, and parent/guardian consent was required to participate. Before data collection, the research was approved by the ethics committee of Hunan Agricultural University (Approval Reference Number: ETHICS 2022107) and was funded by the Hunan Provincial Social Science Foundation in China (Project Reference Number: XSP21YBC310). During the sessions, 405 out of 486 participants completed the study; 50.4% (204) were Male, and 49.6% (201) were Female. All participants provided written informed consent, and the research was conducted according to the Helsinki Declaration. The questionnaires were administered using a paper-and-pencil format to ensure ease of completion and to maintain the tradition of personal engagement in our research methodology.

During pre-intervention (September 2020) and post-intervention (January 2022), trained research assistants administered self-reported questionnaires, including a sociodemographic questionnaire the Morgan-Jinks Student Efficacy Scale (Jinks and Morgan, 1999), Self-Esteem Scale (Rosenberg, 1965; Cheng and Hamid, 1995), and Asher’s Loneliness and Social Dissatisfaction Questionnaire (Asher et al., 1984).

### 2.3 Tools “questionnaires”

#### 2.3.1 Morgan-Jinks student efficacy scale

The Morgan-Jinks Student Efficacy Scale is a tool to determine children’s academic self-efficacy. The scale comprises 30 items, and it uses a 4-point Likert scale that reflects informal language typically used by children. To assess the participating children’s perceived academic self-efficacy at the baseline, we utilized the Chinese version of Jinks and Morgan (1999) Morgan-Jinks Student Efficacy Scale (Zhou et al., 2023). This scale assists practitioners in evaluating students’ beliefs in their academic capabilities by featuring statements such as “I work hard in school” to gage students’ self-efficacy beliefs toward academic success.

#### 2.3.2 Rosenberg’s self-esteem scale

Rosenberg’s self-esteem scale measures an individual’s self-worth and evaluates children’s or adolescents’ perceptions of their worth (Rosenberg, 1965; Cheng and Hamid, 1995). We used the Chinese version of Rosenberg’s (1965) Self-Esteem Scale (RSES-C) to evaluate the self-esteem of the children who participated in the study (Wu et al., 2017). The questionnaire contains ten (10) items on the scale: five (5) items related to positive self-esteem and five (5) items related to self-deprecation, with items such as, “I take a positive attitude toward myself”.

#### 2.3.3 Asher’s loneliness scale

According to Asher et al. (1984), the Children’s Loneliness Scale measures children’s perception of loneliness and social dissatisfaction. We have utilized Asher et al.’s (1984) Child Loneliness Scale (CLS-C) in its Chinese version. The CLS-C has been validated and previously used effectively in research focused on Chinese left-behind children’s loneliness (Chen et al., 2014). The questionnaire consists of 24 items, 16 of which address children’s feelings of loneliness, and the rest focuses on social adequacy (Cassidy and Asher, 1992), employing statements like, “No children play with me.” Each item is rated on a 5-point Likert scale (1 = “totally disagree” to 5 = “totally agree”).

### 2.4 Procedure and intervention

Two artistic sports activities, Aerobic Gymnastics and Latin American dance, were selected between September 2020 and January 2022. One hour and 30 min per lesson, two lessons per week over three semesters, 32 lessons each semester, 96 lessons and 144 h in total. (Three semesters: September 2020 to January 2021, February to June 2021, and September 2021 to January 2022).

Students in six schools (two classes per school) were instructed in the same sub-types of Latin American dance (Samba and Cha-Cha) and Aerobic Gymnastics. At one school, one class taught Latin American dance, and the other taught Aerobic Gymnastics to compare the effectiveness of both activities.

The course consists of three major components: basic dance movements, dance routines for solos and couples, and music understanding. All dance routines were taught to the LBCs during three semesters.

Before the intervention, thirty-six (36) qualified teachers (teachers’ average years of expertise is 6.5, and at least had a minimum of three (3) years of children’s teaching experience) were selected, eighteen (18) each for Aerobic Gymnastics and Latin American Dance. Within each dance style, a random drawing was conducted to divide eighteen (18) teachers into six (6) LBCs’ primary schools. Finally, three (3) places for Aerobic Gymnastics and Latin American Dance teachers were set down in one (1) LBC’s primary school. To avoid the potential influence of teacher effects on the variables’ scores assigned to the children, all teachers involved in the study underwent a unified teaching content learning and training program at the same university before the research commenced. The training program aimed to standardize the instruction and delivery of Aerobic Gymnastics and Latin American dance among all teachers, providing them with detailed guidelines and instructional materials. By equipping the teachers with the necessary expertise to effectively teach these activities to the LBCs, the training program ensured consistent and standardized implementation of the intervention strategies.

Additionally, a team of six PhD-level researchers with backgrounds in psychology was also trained in advance to help collect data and assist the students as they filled out the questionnaire. They utilized non-directive assistance (encouraging LBCs to provide technical support, clarifications, or answer procedural questions if needed, but did not offer any opinions or suggestions that could sway the children’s decisions). They assured them that their responses would remain anonymous and confidential. At the outset and conclusion of the intervention study in six schools, students were asked to complete sociodemographic and three standardized paper-and-pencil questionnaires regarding their academic self-efficacy, self-esteem, and loneliness.

### 2.5 Data analysis

This study examines artistic sports activities interventions (Aerobic Gymnastics and Latin American Dance) to improve students’ academic self-efficacy and self-esteem, reduce loneliness among LBC students in rural areas, and enhance academic performance. Firstly, the Shapiro–Wilks test was chosen for its appropriateness in analyzing the normal distribution of variables within the sample size, considering the participants’ gender and engagement in two distinct artistic sports activities. To ensure the reliability and validity of the questionnaires used in this study, both Cronbach’s alpha (α) and the Kaiser-Meyer-Olkin (KMO) coefficient were calculated. Furthermore, the relationships among academic self-efficacy, loneliness, and self-esteem (including its sub-dimensions, Positive Esteem and Self-Deprecation) were initially explored using Pearson correlation analysis (*R*-values) to identify the strength and direction of these associations.

Then, independent samples t-tests were conducted to compare differences across various demographic variables, including gender. Additionally, paired samples t-tests were utilized to examine the differences in psychological indicators related to artistic sports activities before and after the intervention. Following this, Pearson correlation analysis was employed to determine the relationships among the variables (i.e., LCB’s academic self-efficacy, self-esteem, and loneliness), laying the groundwork for testing mediator and moderator effects (Ren and Li, 2020). This clarification specifies the types of t-tests performed, enhancing the reader’s understanding of the statistical methods used.

Meanwhile, mediation analyses were conducted using the pre-interventional data, which included the psychometric variables of “students’ academic self-efficacy,” “self-esteem,” and “loneliness.” These analyses aimed to explore whether loneliness served as a mediator between self-esteem and academic self-efficacy among left-behind children (LBCs), with the pre-intervention data chosen for its potential to reflect the actual mental state of LBCs better after the data collection had ended. The findings from these analyses will provide insights into the interplay between self-esteem, loneliness, and academic self-efficacy among LBCs, shedding light on the mechanisms that may influence their academic performance.

Multiple linear regression was employed to assess the role of loneliness as a mediator between self-esteem and student’s academic self-efficacy, using bootstrapping to test the significance of the indirect effect. For this purpose, 95% bias-corrected confidence intervals were generated through Bootstrap sampling. An indirect effect was considered significant if the 95% bias-corrected confidence interval did not include zero. The statistical analyses were performed using SPSS 28.0, including the PROCESS macro by Hayes (2017) for bootstrapping procedures essential for testing the significance of indirect effects in mediation analysis, which ensuring a precise and reliable examination of the hypothesized relationships.

## 3 Results

### 3.1 Participant characteristics and reliability-validity of scales

During the sessions, 83.4% (405 out of 486) participants (9 to 13 years old) completed the study; 50.4% (204) were Male, and 49.6% (201) were Female. The 16.6% withdrew for various reasons, including lack of interest and shyness. The Shapiro–Wilk test results indicated the data were normally distributed (*p* values >0.05). Using Cronbach’s alpha (α) and Kaiser-Meyer-Olkin (KMO) coefficients, the study showed good reliability and validity of the overall scales. Morgan and Jink’s children’s perceived academic self-efficacy scale are both 0.75 (Good) and 0.79 (Average suitability), Rosenberg’s self-esteem scale, Cronbach’s alpha coefficient is 0.83 (Good), while the KMO coefficient is 0.9 (Suitable), Asher children’s loneliness scale, Cronbach’s alpha coefficient in this study is 0.71 (Good), and the KMO coefficient is 0.78 (Average suitability).

### 3.2 Impact of artistic sports activities among left-behind children

Pre- and Post-student academic self-efficacy have a *p* < 0.01 (*t* = −39.22, *p* = 0.000), and the specific comparison difference shows that Pre-student academic self-efficacy (62.70 ± 10.48) has a significantly lower average value than Post-student academic self-efficacy (90.60 ± 9.41); while the specific comparison difference shows that Pre- Self-Esteem (15.42 ± 3.21) is significantly lower than Post- Self-Esteem (28.79 ± 4.91), with a level of *p* < 0.01 significance between the two tests (*t* = −45.38, *p* = 0.000); also A *p* < 0.01 level of significance exists between Pre- and Post-Loneliness (*t* = 6.69, *p* = 0.000), the specific comparison difference indicates a significantly higher average value for Pre-Loneliness (35.12 ± 8.02) than Post-Loneliness (33.36 ± 7.72). All data are reported in Table 2, which evidence that artistic sports activities significantly impact all LBC’s psychological variables of student academic self-efficacy, self-esteem, and loneliness.

**Table 2:** Pre-test and post-test of artistic sports activities group intervention (*N* = 405).

Group	Paired statistics (Mean ± SD)		Paired mean	*T*	*p*-value
	Pre-test	Post-test			
Academic self-efficacy	62.70 ± 10.48	90.60 ± 9.41	−27.90	−39.220	0.000**
Male	63.44 ± 10.25	89.31 ± 9.75	−25.87	−25.897	0.000**
Female	61.95 ± 10.67	91.91 ± 8.88	−29.96	−30.118	0.000**
Self-Esteem	15.42 ± 3.21	28.79 ± 4.91	−13.37	−45.380	0.000**
Male	15.22 ± 3.01	28.46 ± 4.85	−13.24	−32.690	0.000**
Female	15.63 ± 3.40	29.13 ± 4.97	−13.50	−31.463	0.000**
loneliness scale	35.12 ± 8.02	33.36 ± 7.72	1.76	6.693	0.000**
Male	34.63 ± 8.27	33.46 ± 7.59	1.17	4.098	0.000**
Female	35.67 ± 7.71	33.25 ± 7.88	2.41	5.350	0.000**

Table shows the general means (Mean), standard deviations (SD). Paired T-tests to exam differences Pre-, Post- Test. **Indicates a significant relationship between the variables. *Significant (*p* < 0.05) **Significant (*p* < 0.01).

In terms of different artistic sports activities there was also a significant positive impact on all LBC’s psychological variables in different artistic sports activities (Aerobic Gymnastics and Latin American Dance) between pre-test and post-test (*p* < 0.01). In the Aerobic Gymnastics intervention group, post-test scores for self-esteem and student academic self-efficacy were significantly higher than pre-student academic self-efficacy scores (*t* = −28.77, *p* < 0.01) and pre-self-esteem (*t* = −33.12, *p* < 0.01), also post-test loneliness scores were significantly lower than pre-test levels (*t* = 4.73, *p* < 0.01). In the Latin American Dance group intervention group, post-test scores for self-esteem and student academic self-efficacy were significantly higher than pre-student academic self-efficacy scores (*t* = −26.72, *p* < 0.01) and pre-self-esteem (*t* = −31.13, *p* < 0.01), also post-test loneliness scores were significantly lower than pre-test levels (*t* = 4.73, *p* < 0.01). Data are reported in Table 3.

**Table 3:** Pre-test and post-test of aerobic gymnastics group/Latin American dance group intervention (*N* = 405).

Group		Paired statistics (Mean ± SD)		Paired mean	*T*	*p*-value
		Pre-test	Post-test			
Aerobic Gymnastics	Academic self-efficacy	62.34 ± 10.28	90.73 ± 9.53	−28.39	−28.77	0.000**
	Self-Esteem	15.26 ± 3.05	28.52 ± 4.79	−13.26	−33.12	0.000**
	Loneliness scale	34.77 ± 7.90	33.01 ± 7.55	1.76	4.73	0.000**
Latin American Dance	Academic self-efficacy	63.05 ± 10.68	90.47 ± 9.31	−27.41	−26.72	0.000**
	Self-Esteem	15.58 ± 3.37	29.06 ± 5.04	−13.48	−31.13	0.000**
	Loneliness scale	35.47 ± 8.13	33.71 ± 7.89	1.76	4.73	0.000**

Table shows the general means (Mean), and standard deviations (SD). Paired T-tests to exam differences Pre-, Post- Test. **indicates a significant relationship between the variables. *Significant (*p* < 0.05); **Significant (*p* < 0.01).

### 3.3 Self-efficacy, self-esteem, and loneliness

Table 4 provides correlation and statistical information for the main variables between the five items, including self-esteem with its two sub-dimensions (positive esteem and self-deprecation), student academic self-efficacy, and loneliness. The R-value indicates the degree of correlation between each variable by identifying strong or weak correlations. The specific analysis shows that student academic self-efficacy is strongly positively correlated with self-esteem (*r* = 0.117, *p* < 0.05) and strongly negatively correlated with loneliness (*r* = −0.276), with a significant statistical correlation (*p* < 0.01). A significant positive correlation was also found between student academic self-efficacy and the two self-esteem sub-dimensions “positive self-esteem (*r* = 0.105, *p* < 0.05)” and “self-deprecation (*r* = 0.109, *p* < 0.05).” Additionally, self-esteem exhibited a significant negative correlation with loneliness (*r* = −0.168, *p* < 0.01), as well as loneliness demonstrated a significant negative correlation with two sub-dimensions of self-esteem, “positive self-esteem” (*r* = −0.134, *p* < 0.01) and “self-deprecation” (*r* = −0.176, *p* < 0.01).

**Table 4:** Descriptive statistics and Pearson correlation coefficient table (*N* = 405).

	Mean	SD	1	2	3	4	5
Age	10.510	1.360					
1. Self-Esteem	28.790	4.914	1				
2. Positive Esteem	15.800	2.685	0.920**	1			
3. Self-Deprecation	12.990	2.662	0.918**	0.690**	1		
4. Academic Self-Efficacy	90.600	9.407	0.117*	0.105*	0.109*	1	
5. Loneliness	33.363	7.719	−0.168**	−0.134**	−0.176**	−0.276**	1

All tests were two-tailed. This table shows the general means (Mean), standard deviations (SD), and correlations of the six major variables. **Indicates a significant correlation between the variables, which obtains between all the variables. *Significant (*p* < 0.05); **Significant (*p* < 0.01).

### 3.4 Mediator’s role of loneliness

As shown in Table 5, study results indicated that in Model 1: self-esteem is an effective predictor of student academic self-efficacy (*β* = 0.117, *p* < 0.05). Consequently, in Model 2, the study employed loneliness as the dependent variable and self-esteem as the predictor variable. It was found that self-esteem had an effect on the prediction of Loneliness (*β* = −0.168, *p* < 0.01). Furthermore, in Model 3, using student academic self-efficacy as the dependent variable and self-esteem and loneliness as the independent variables, the results of the multiple linear regression analysis revealed that when the loneliness variable was included, the regression coefficient of self-esteem for student academic self-efficacy decreased (Tables 5, 6) displays total effect (*c* = 0.223) and direct effect (*c*’ = 0.138) and significant (*β* = 0.072, *p* < 0.01). As well the results show that loneliness can effectively predict student academic self-efficacy (*β* = −0.264, *p* < 0.01).

**Table 5:** Direct effect of loneliness on self-esteem and academic self-efficacy (*N* = 405).

Independent variable	Dependent variable	*β*	*T*	*R* ^2^	Adjusted *R*^2^	F
Self-esteem	Academic self-efficacy	0.117	2.358	0.014	0.011	5.562*
Self-esteem	Loneliness	−0.168	−3.429	0.028	0.026	11.755**
Self-esteem	Academic self-efficacy	0.072	1.489	0.081	0.077	17.817**
Loneliness		−0.264	−5.448			

Model 1, Regression model of “Academic self-efficacy” to “General Self-Efficacy” Model 2, Regression model of “Academic self-efficacy” to “Self-Esteem” Model 3, Regression model of “Academic self-efficacy” with the mediating variable “Self-Esteem” to “General Self-Efficacy”. *Significant (*p* < 0.05). **Significant (*p* < 0.01).

**Table 6:** Indirect effect results.

Item	B(c) TE	a	b	a*b ME	B(c’) DE	a*b (95% Boot CI)
Self-esteem = > Loneliness = > Academic self-efficacy						
	0.223*	−0.264**	−0.322**	0.085	0.138 **	0.012 ~ 0.088

“B(c)”: Represents the regression coefficient of “Academic self-efficacy” to “General Self-Efficacy” (no mediator Self-Esteem in the model), i.e., Total Effect (TE). “B(c’)”: Represents the regression coefficient of “Academic self-efficacy” to “General Self-Efficacy” (included mediator Self-Esteem in the model), i.e., Direct Effect (DE). “a”: Represents the regression coefficient of “Academic self-efficacy” to “Self-Esteem.” “b”: Represents the regression coefficient “Self-Esteem” to “General Self-Efficacy.” “a*b”: Represents the product of a and b, i.e., Mediation effect (ME). 95% Boot CI represents the 95% confidence interval calculated by Bootstrap sampling, if the interval does not include 0, it means significant. “Effect Ratio”: If it is a complete mediation, the effect ratio is 100%; If it is a partial mediation, the formula is: a*b/c; If the mediation effect is not significant, the effect ratio is 0%. *Significant (*p* < 0.05). **Significant (*p* < 0.01).

Moreover, Table 6 shows that indirect of self-esteem on academic self-efficacy via decreased loneliness was significant for 95% Boot CI (the interval is 0.012 ~ 0.088, does not include 0), and the mediation effect was 0.085, which demonstrates that loneliness mediates self-esteem and student academic self-efficacy. The findings above indicate that loneliness influences the relationship between academic self-efficacy and self-esteem among LBC students. Loneliness is perceived as a mediator.

**Figure 1:**
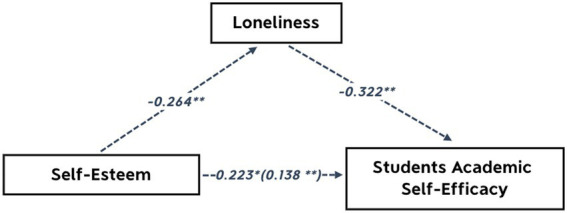
Model of mediation effect (indirect effects). **Significant (*p* < 0.01).

To simplify the model, the mediating effect of loneliness in the multiple linear regression model is shown in Figure 1.

## 4 Discussion

### 4.1 Summary of findings

Among Chinese LBCs, the results supported the hypothesis that artistic sports activities (Aerobic Gymnastics and Latin American Dance) intervention can significantly boost academic performance by improving students’ academic self-efficacy and self-esteem and decreasing loneliness. Moreover, based on the preconditions of loneliness being strongly negatively correlated with LBC’s self-esteem and academic self-efficacy, the findings also supported the hypothesis that loneliness was a mediator of the relationship between academic self-efficacy and self-esteem among LBCs.

### 4.2 Highlights and implications

#### 4.2.1 Impact of artistic sports activities among left-behind children

The data reveals that the psychological variables of self-esteem and academic self-efficacy significantly improved after Aerobic Gymnastics and Latin American dance practice. The result is consistent with previous studies on Aerobic Gymnastics activities, which show that aerobic gymnastics cannot only have a positive effect on students’ academic performance (Marino, 2010; Lucky, 2016) but also improve their self-esteem (Marino, 2010), after a study Marino (2010) in which 64 students (8–10 years old) participated in an aerobic dance intervention once a week for 8 weeks during their physical education classes for 40 min. However, research on Latin American dance’s impact on academic self-efficacy is limited. Some studies indicate that dance participation may improve students’ academic performance, such as Higueras-Fresnillo et al. (2016) study, 714 young girls aged 11.83 ± 2.50 who participated in dance scored significantly higher in academic performance (Mathematics and Language) than those who did not. This supports our finding that Latin American dance positively impacts LBCs’ academic self-efficacy.

Additionally, a study by Sanchez (2020) found that Latin American dance (salsa) promotes compassion and healthy self-esteem in children, and Yuan (2021) concluded that Latin American dance not only has a positive effect on self-esteem and its dimensions but also can enhance learners’ mental health through better social development. A systematic review by Park (2021) found that dance movement (dance-related activities) could improve children’s self-esteem. Based on this study, research data indicate that Latin American dance interventions in LBCs can effectively improve their academic self-efficacy.

The results highlight that artistic sports activities can help LBCs become more socially connected and create peer relationships to reduce loneliness, improve a student’s self-esteem and enhance academic self-efficacy, resulting in better academic performance. Studies on the impact of artistic sports activities (Aerobic Gymnastics and Latin American Dance) on loneliness are relatively scarce. Some studies indicate that participation in sports may reduce feelings of loneliness among children, as Page et al. (1992) found that children (ages 6 to 11) who participated in sports reported lower levels of loneliness, and Weiss and Smith (2002) found that participating in sports can reduce feelings of loneliness by providing positive peer interaction. This is probably why artistic sports activities positively impact the loneliness of LBCs.

#### 4.2.2 Mediator’s role of loneliness

This is the first research concerning whether loneliness mediates self-esteem and academic self-efficacy in LBCs. Based on the theoretical explanation in the introduction section revealed that individuals’ mental states are influenced by a combination of self-influence factors and social systems (family and peers). Therefore, the relationships individuals have with their family and peers have the potential to impact self-influence factors, such as self-esteem and academic self-efficacy, in which loneliness is one of the impacted factors that family and peers influence.

Based on the findings, loneliness mediated the relationship between academic self-efficacy and self-esteem among LBCs. There are two reasons to support this result. Firstly, student academic performance is strongly negatively correlated with loneliness after studying 438 elementary school students from 9 to 13 in China (Lian-Yun, 2013), and loneliness scores were negatively correlated with academic self-efficacy after investigating school children’s drinking behavior (mean age 13.5 years) (McKay et al., 2017). Secondly, loneliness among LBCs is closely related to the absence of parental support (Li et al., 2021) and friendship (Zhang et al., 2021).

### 4.3 Conclusion

The results show that Artistic Sports Activities (Aerobic Gymnastics and Latin American Dance) intervention can effectively and positively improve each psychometric test variable of students’ academic self-efficacy, self-esteem, and loneliness among LBCs in Chinese rural areas to boost students’ academic performance, which matched the research aim of this study. The results also show that loneliness mediated the relationship between academic self-efficacy and self-esteem among LBCs.

This study offers new insights into how artistic sports like Aerobic Gymnastics and Latin American Dance affect Left-Behind Children (LBCs) in three main areas: academic confidence (self-efficacy), self-esteem, and feelings of loneliness. Our work especially shows the psychological benefits of these artistic sports. We found that loneliness impacts LBCs’ feelings about themselves and their academic abilities and is crucial in linking the two. This helps us understand better how emotions and social connections influence learning and self-view among LBCs. For educators and those working with children, our findings suggest a practical way to help LBCs feel better about themselves and do better in school. By including Aerobic Gymnastics and Latin American Dance in school activities, we can address their academic needs and improve their mental health and social connections. This approach could make a significant difference for LBCs, offering them a more supportive and enriching school experience.

### 4.4 Limitations and future research directions

Several limitations were noticed in this study. The study examined the effects of artistic sports activities (Aerobic Gymnastics and Latin American Dance) on the academic performance of LBC, which considered the two styles as interventions. It would be more valuable to explain the impacts of different artistic sports activity styles, such as Figure Skating, ballroom dance, or Synchronized Swimming. (ii) Intervention was carried out only three semesters (one and a half years). Thus, a more comprehensive analysis of the long-term effects of our study is necessary. Meanwhile, this study did not include a control group, a limitation that merits acknowledgement. While employing a within-person/repeated measure intervention design allowed for the observation of significant differences in study outcome variables at pre- and post-intervention phases, the absence of a control group raises uncertainties regarding the extent to which observed changes can be attributed directly to the intervention, as opposed to other unobserved variables or changes occurring during the study period. Future iterations of this research will benefit from incorporating a control group to enhance the clarity and reliability of the results obtained.

For future research, these findings address a previous void in the literature, emphasizing the potential of artistic sports activities to reinforce psychological well-being among LBC groups. In the context of school psychology, this implies that interventions aimed at elevating self-esteem and mitigating feelings of loneliness can further bolster the academic self-efficacy of LBCs, thereby improving their academic performance. The increased social connectivity and peer relationship development stemming from these activities highlight their value in mitigating loneliness, thus boosting students’ self-esteem and academic self-efficacy, resulting in better academic performance. Educators, school psychologists, researchers, and practitioners should consider integrating such artistic sports activities within the school curriculum, whether in PE or art classes, to harness these psychological benefits for LBCs. Future research might explore diverse artistic sports activity formats to curate sustainable interventions and also delve into a broader spectrum of psychological metrics, like social anxiety, to yield more holistic insights into the efficacy of these artistic sports activity interventions.

## Data availability statement

Due to ethics restrictions, the data are not publicly available. The data can be obtained from the corresponding author based on a reasonable request for research purposes. Requests to access the datasets should be directed to Zhouyutao@hut.edu.cn.

## Ethics statement

The studies involving humans were approved by Hunan Agricultural University (Approval Reference Number: ETHICS 2022107). The studies were conducted in accordance with the local legislation and institutional requirements. Written informed consent for participation in this study was provided by the participants’ legal guardians/next of kin.

## Author contributions

YZ: Writing – original draft, Writing – review & editing. FF: Conceptualization, Data curation, Formal analysis, Methodology, Software, Writing – review & editing, Writing – original draft. CF: Conceptualization, Formal analysis, Funding acquisition, Methodology, Resources, Software, Validation, Writing – original draft, Writing – review & editing.
