# Supplementary material for: Artistic sports activities effectiveness for enhancing students’ academic performance among left-behind children: mediating effects of loneliness
Source: Front Psychol. 2024 May 6;15:1366501. doi: 10.3389/fpsyg.2024.1366501 (PMC11104499; doi:10.3389/fpsyg.2024.1366501)
Supplement: Supplementary file 1 [file Table_1.DOCX]

Supplementary Material - Tables

Artistic Sports Activities Effectiveness for Enhancing Students Academic Performance Among Left-Behind Children: Mediating Eﬀects of Loneliness

**Yutao Zhou ^1,3,^*, Francesco Vincenzo Ferraro ^2,3^ , Chengwen Fan^1,3^**

*^1^ Physical Education Institute, Hunan University of Technology, Zhuzhou 412000, China*

*^2^ School of Human Sciences, University of Derby, Kedleston Road, Derby DE22 1GB, UK*

*^3^ Hunan Research Centre for Excellence in Fitness, Health & Performance, Zhuzhou 412000, China*

**^*^Correspondence:** Yutao Zhou [Zhouyutao@hut.edu.cn](mailto:Zhouyutao@hut.edu.cn)

# Supplementary Tables/Figures

# Table 1. Overview of Psychometric Variables Influencing Academic Performance

| Psychometric Variable | Definition | Influence on Academic Performance |
| --- | --- | --- |
| Students’ Academic Self-Efficacy | A student's belief in their ability to meet the demands of their academic environment (Fife et al., 2011). | Significant influence on predicting academic resilience (Bandura, 1997). Facilitates the development of academic goal-setting skills (Locke and Latham, 2002) and impacts academic performance (Honicke and Broadbent, 2016). |
| Self-Esteem | A psychological state corresponds to a sense of integrity in understanding one's capabilities and value (Saha, 2018). | Mediates the relationship between parent-child relationships and academic stress (Mulyadi et al., 2016). Strongly correlates with academic performance and teacher support (Liu et al., 2019). |
| Loneliness | An unpleasant emotion of discrepancy between desired and experienced interpersonal relationships (Perlman and Peplau, 1982), is typically experienced by LBC (Shen et al., 2015). | Associated with a lack of family connections (Cacioppo et al., 2015), and correlated with individual problems among LBC, such as emotional problems (e.g., social anxiety) (Li et al., 2020), psychological problems (e.g., self-esteem) (Wei and Huang, 2019), and education-related problems (e.g., student academic performance or achievement) (Wang et al., 2021b). |

| **Table 2.** Pre-test and Post-test of Artistic Sports Activities Group Intervention (N=405) | | | | | |
| --- | --- | --- | --- | --- | --- |
| **Group** | **Paired Statistics (Mean ± SD)** | | **Paired Mean** | **T** | **p** |
|  | **Pre-Test** | **Post-Test** |  |  |  |
| Academic self-efficacy | 62.70±10.48 | 90.60±9.41 | -27.90 | -39.220 | 0.000** |
| Male | 63.44±10.25 | 89.31±9.75 | -25.87 | -25.897 | 0.000** |
| Female | 61.95±10.67 | 91.91±8.88 | -29.96 | -30.118 | 0.000** |
| Self-Esteem | 15.42±3.21 | 28.79±4.91 | -13.37 | -45.380 | 0.000** |
| Male | 15.22±3.01 | 28.46±4.85 | -13.24 | -32.690 | 0.000** |
| Female | 15.63±3.40 | 29.13±4.97 | -13.50 | -31.463 | 0.000** |
| loneliness scale | 35.12±8.02 | 33.36±7.72 | 1.76 | 6.693 | 0.000** |
| Male | 34.63±8.27 | 33.46±7.59 | 1.17 | 4.098 | 0.000** |
| Female | 35.67±7.71 | 33.25±7.88 | 2.41 | 5.350 | 0.000** |
| Table shows the general means (Mean), standard deviations (SD). Paired T-tests to exam differences Pre-, Post- Test.  ** indicates a significant relationship between the variables. * Significant (p<0.05) ** Significant (p<0.01) | | | | | |

| **Table 3.** Pre-test and Post-test of Aerobic Gymnastics Group / Latin American Dance Group Intervention (N=405) | | | | | | |  |
| --- | --- | --- | --- | --- | --- | --- | --- |
| **Group** | | **Paired Statistics (Mean ± SD)** | | **Paired Mean** | **T** | **p** | |
|  |  | **Pre-Test** | **Post-Test** |  |  |  |  |
| Aerobic Gymnastics | Academic self-efficacy | 62.34±10.28 | 90.73±9.53 | -28.39 | -28.77 | 0.000** | |
|  | Self-Esteem | 15.26±3.05 | 28.52±4.79 | -13.26 | -33.12 | 0.000** | |
|  | loneliness scale | 34.77±7.90 | 33.01±7.55 | 1.76 | 4.73 | 0.000** | |
| Latin American Dance | Academic self-efficacy | 63.05±10.68 | 90.47±9.31 | -27.41 | -26.72 | 0.000** | |
|  | Self-Esteem | 15.58±3.37 | 29.06±5.04 | -13.48 | -31.13 | 0.000** | |
|  | loneliness scale | 35.47±8.13 | 33.71±7.89 | 1.76 | 4.73 | 0.000** | |

Table shows the general means (Mean), and standard deviations (SD). Paired T-tests to exam differences Pre-, Post- Test.

** indicates a significant relationship between the variables. * Significant (p<0.05) ** Significant (p<0.01)

| **Table 4.** Descriptive Statistics and Pearson Correlation Coefficient Table (N=405) | | | | | | | |
| --- | --- | --- | --- | --- | --- | --- | --- |
|  | **Mean** | **SD** | **1** | **2** | **3** | **4** | **5** |
| Age | 10.510 | 1.360 |  |  |  |  |  |
| 1. Self-Esteem | 28.790 | 4.914 | 1 |  |  |  |  |
| 2. Positive Esteem | 15.800 | 2.685 | 0.920** | 1 |  |  |  |
| 3. Self-Deprecation | 12.990 | 2.662 | 0.918** | 0.690** | 1 |  |  |
| 4. Academic Self-Efficacy | 90.600 | 9.407 | 0.117* | 0.105* | 0.109* | 1 |  |
| 5. Loneliness | 33.363 | 7.719 | -0.168** | -0.134** | -0.176** | -0.276** | 1 |
| All tests were two-tailed. This table shows the general means (Mean), standard deviations (SD), and correlations of the six major variables. ** indicates a significant correlation between the variables, which obtains between all the variables.  * Significant (p<0.05) ** Significant (p<0.01) | | | | | | | |

| **Table 5.** Direct Effect of Loneliness on Self-Esteem and Academic Self-Efficacy (N=405) | | | | | | |
| --- | --- | --- | --- | --- | --- | --- |
| **Independent variable** | **Dependent variable** | **β** | **T** | **R²** | **Adjusted R²** | **F** |
| Self-Esteem | Academic Self-Efficacy | 0.117 | 2.358 | 0.014 | 0.011 | 5.562 * |
| Self-Esteem | Loneliness | -0.168 | -3.429 | 0.028 | 0.026 | 11.755 ** |
| Self-Esteem | Academic Self-Efficacy | 0.072 | 1.489 | 0.081 | 0.077 | 17.817 ** |
| Loneliness |  | -0.264 | -5.448 |  |  |  |
| Model 1, Regression model of “Academic self-efficacy” to “General Self-Efficacy” Model 2, Regression model of “Academic self-efficacy” to “Self-Esteem” Model 3, Regression model of “Academic self-efficacy” with the mediating variable “Self-Esteem” to “General Self-Efficacy”. * Significant (p<0.05) ** Significant (p<0.01) | | | | | | |

| **Table 6.** Indirect Effect Results | | | | | | |
| --- | --- | --- | --- | --- | --- | --- |
| **Item** | **B(c)**  **TE** | **a** | **b** | **a*b**  **ME** | **B(c’)**  **DE** | **a*b (95% Boot CI)** |
| Self-Esteem  => Loneliness  => Academic Self-Efficacy | 0.223* | -0.264** | -0.322** | 0.085 | 0.138 ** | 0.012 ~ 0.088 |
| “B(c)”: Represents the regression coefficient of “Academic self-efficacy” to “General Self-Efficacy” (no mediator Self-Esteem in the model), i.e., Total Effect (TE). “B(c’)”: Represents the regression coefficient of “Academic self-efficacy” to “General Self-Efficacy” (included mediator Self-Esteem in the model), i.e., Direct Effect (DE). “a”: Represents the regression coefficient of “Academic self-efficacy” to “Self-Esteem”. “b”: Represents the regression coefficient “Self-Esteem” to “General Self-Efficacy”. “a*b”: Represents the product of a and b, i.e., Mediation effect (ME). 95% Boot CI represents the 95% confidence interval calculated by Bootstrap sampling, if the interval does not include 0, it means significant. “Effect Ratio”: If it is a complete mediation, the effect ratio is 100%; If it is a partial mediation, the formula is: a*b/c; If the mediation effect is not significant, the effect ratio is 0% * Significant (p<0.05) ** Significant (p<0.01) | | | | | | |
